# Higher Balanced Solution Use Is Associated With Less Severe Kidney Injury And Mortality: A Mimic 2 Analysis

**DOI:** 10.1186/2197-425X-3-S1-A15

**Published:** 2015-10-01

**Authors:** FG Zampieri, OT Ranzani, IDS Martins, AB Libório

**Affiliations:** Hospital das Clínicas, Intensive Care Unit, São Paulo, Brazil; Hospital Alemão Oswaldo Cruz, Intensive Care Unit, São Paulo, Brazil; Hospital das Clínicas, University of São Paulo, Respiratory Intensive Care Unit, São Paulo, Brazil; Faculdade de Medicina da Universidade do Ceará, Programa de Pós-graduação em Ciência Médicas, Fortaleza, Brazil

## Introduction

Balanced solutions may be associated with lower morbidity in critical illness in comparison with chloride-rich solutions, including acute kidney injury (AKI). Lactated ringer (LR) is one of most widely used balanced solutions. Nevertheless, it is unclear if fluid mix during the first days of ICU admission influences the development of AKI after controlling for other relevant confounders such as fluid balance, illness severity and comorbidities.

## Objectives

To assess the association between the higher percentages of fluid administered as LR (%LR) in the first 48 hours of ICU stay and the risk of developing severe AKI (KDIGO II/III) during the first 7 of ICU stay. As secondary endpoint, we assessed the impact of %LR in hospital mortality.

## Methods

Data were extracted from MIMIC-2 database, which contain information from more than 32,000 patients. All infused volume of LR, NaCl 0.45%, NaCl 0.9%, NaCl 3% and glucose 5% were retrieved from the database. %LR was calculated as total amount of LR infused / total amount of fluid infused. Data were adjusted for illness severity (SAPS 1), fluid balance, ICU type, age, gender and major comorbidities. Patients with chronic kidney disease (admission creatinine higher than 4 mg/dL) and with short ( < 48h) ICU stay were excluded. Analysis was performed through logistic regression.

## Results

**Figure 1:**
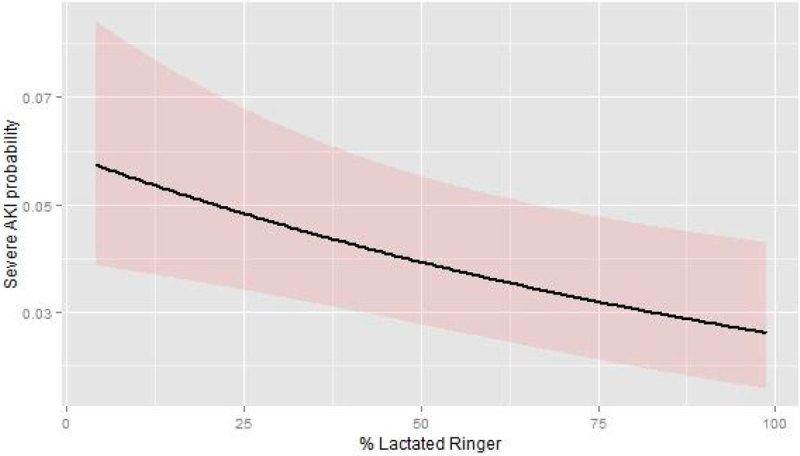
Figure 1

After all inclusion criteria were required, 4295 patients remained in the final analysis and 334 (7%) patients developed severe AKI and 352 (8%) died during hospital stay. Increasing %LR was associated with lower incidence of severe AKI (shown in Figure 1, OR 0.91; 95% CI 0.86-0.97 for each 10% increase) and with lower hospital mortality (OR 0.82, 95% CI 0.77-0.87 for each 10% increase).

Other factors associated with severe AKI development were SAPS (OR 1.11 for each point increase, 95% CI 1.08-1.15), upper quartile of fluid balance (OR 3.08, 95% CI 2.12-4.51), presence of liver disease (OR 2.20, 95% CI 1.31-3.69), presence of heart failure (OR 1.77, 95% CI 1.31-2.38) and admission to a surgical ICU (OR 2.06, 95% CI 1.47-2.88).

## Conclusions

Higher %LR is independently associated with lower AKI and lower mortality in a wide population of critically ill patients. These findings corroborate to the concept that balanced solutions should be the preferred fluid type in the critically ill.
